# Nomenclature for Factors of the HLA System, Update January, February and March 2025

**DOI:** 10.1111/iji.12713

**Published:** 2025-04-28

1

The following sequences have been submitted to the Nomenclature Committee since the October, November and December 2024 nomenclature update (Marsh, 2025) and, following agreed policy, have been assigned official allele designations (Marsh et al. 2010). Full details of all sequences will be published in a forthcoming report.

Below are listed the newly assigned sequences (Table 1) and confirmations of previously reported sequences (Table 2). The accession number of each sequence is given and these can be used to retrieve the sequence files from the EMBL, GenBank or DDBJ data libraries. Although accession numbers have been assigned by the data‐libraries and most sequences are already available, there is still the possibility that an author may not yet have allowed the sequence to be released; in such a case you will have to contact the submitting author directly. Additional information pertaining to new sequences is often included in the publications describing these alleles; a listing of recent publications that describe new HLA sequences is given in Table 3.

**TABLE 1:** New sequences.

HLA Allele	Cell identification	Accession number	Submitting author
A*01:01:01:121	232624	PV083176	Dr Vasiliki Kitsiou, Athens, Greece
A*01:01:162	017386	PV037023	Dr Lia Mele, Alessandria, Italy
A*01:454:02	KSR201439	PQ665512	Dr Maria Loginova, Kirov, Russia
A*01:479	HG00055356	PQ801759	Histogenetics, Ossining, USA
A*01:480	HG00055393	PQ881810	Histogenetics, Ossining, USA
A*02:01:01:258	HG00055315	PQ736461	Histogenetics, Ossining, USA
A*02:55:02	KSR157495	PQ665513	Dr Maria Loginova, Kirov, Russia
A*02:76:04	1231382	OZ199529	Miss Kristin Kipper, Martinsried, Germany
A*02:1207	2023112242	MZ714558	Dr Mingrui Huo, Beijing, China
A*02:1208N	HG00051318	OR452118	Histogenetics, Ossining, USA
A*02:1209N	703994	OZ199515	Miss Kristin Kipper, Martinsried, Germany
A*02:1210	H170897	PQ816216	Dr Dan Zhang, Beijing, China
A*02:1211	HG00055358	PQ820455	Histogenetics, Ossining, USA
A*02:1212	1805192	PQ382550	Prof Maria Gerbase De Lima, Sao Paulo, Brazil
A*02:1213	YSW‐001	PQ815855	Dr Cheng Yan Fan, Beijing, China
A*02:1214N	796263	OZ199530	Miss Kristin Kipper, Martinsried, Germany
A*02:1215	944085	OZ199521	Miss Kristin Kipper, Martinsried, Germany
A*02:1216	1022725	OZ199523	Miss Kristin Kipper, Martinsried, Germany
A*02:1217	1080438	OZ199525	Miss Kristin Kipper, Martinsried, Germany
A*02:1218	1088887	OZ199526	Miss Kristin Kipper, Martinsried, Germany
A*02:1219N	1818962	PQ349841	Prof Maria Gerbase De Lima, Sao Paulo, Brazil
A*02:1220	HG00055394	PQ881811	Histogenetics, Ossining, USA
A*02:1221Q	SMC018	LC859410	Dr Eunsuk Kang, Seoul, South Korea
A*02:1222N	HG00055431	PV174108	Histogenetics, Ossining, USA
A*03:01:01:124	HG00055355	PQ778466	Histogenetics, Ossining, USA
A*03:01:01:125	24404915	PQ857590	Dr Luis A Marin, Albacete, Spain
A*03:01:133	6211995‐EVS	PQ672531	Dr Jeane Visentainer, Maringa, Brazil
A*03:505	KSR152382	PQ665514	Dr Maria Loginova, Kirov, Russia
A*03:506	852922	OZ199520	Miss Kristin Kipper, Martinsried, Germany
A*03:507	1071967	OZ199524	Miss Kristin Kipper, Martinsried, Germany
A*03:508	HG00055398	PQ881815	Histogenetics, Ossining, USA
A*11:01:01:94	NGS1354	PQ821711	Dr Meenaskshi Singh, Navi Mumbai, India
A*11:01:134	HG00050451	OP314559	Histogenetics, Ossining, USA
A*11:01:135	42448158	PQ817304	Dr Romain Ferru‐Clement, Poitiers, France
A*11:02:09	21QB004050	OZ121613	Dr Janette Kwok, Pokfulam Road, Hong Kong
A*11:487	HG00055401	PV037250	Histogenetics, Ossining, USA
A*11:488	HG00055397	PQ881814	Histogenetics, Ossining, USA
A*11:489	HN2411	PQ412961	Dr Zheng Liu, Zhengzhou, China
A*11:490	HG00055433	PV174110	Histogenetics, Ossining, USA
A*23:01:01:35	24405006	PQ998756	Dr Luis A Marin, Albacete, Spain
A*23:01:42	24227773	PV021448	Dr Daniel Fuerst, Ulm, Germany
A*23:150	KSR218449	PQ665515	Dr Maria Loginova, Kirov, Russia
A*24:02:179	KSR226865	PQ753112	Dr Maria Loginova, Kirov, Russia
A*24:649	DE2‐380	PQ486538	Dr Zhangxiang Wanyan, Kunming, China
A*24:650	C00062314146242	PQ336759	Dr William Lemieux, Saint‐Laurent, Canada
A*24:651N	748351	OZ199516	Miss Kristin Kipper, Martinsried, Germany
A*24:652	1814756	PQ466180	Prof Maria Gerbase De Lima, Sao Paulo, Brazil
A*24:653	816419	OZ199518	Miss Kristin Kipper, Martinsried, Germany
A*24:654	1792517	PQ466171	Prof Maria Gerbase De Lima, Sao Paulo, Brazil
A*25:01:23	KSR173223	PQ753113	Dr Maria Loginova, Kirov, Russia
A*26:03:04	1807357	PQ466172	Prof Maria Gerbase De Lima, Sao Paulo, Brazil
A*26:254	2400827	PQ801844	Mrs Malgorzata Kosierb, New York, USA
A*26:255	KSR187230	PV219215	Dr Maria Loginova, Kirov, Russia
A*29:02:40	HG00055400	PV037249	Histogenetics, Ossining, USA
A*29:201	1169087	OZ199528	Miss Kristin Kipper, Martinsried, Germany
A*30:04:05	1816573	PQ382548	Prof Maria Gerbase De Lima, Sao Paulo, Brazil
A*30:238	HWF‐001	PQ815854	Dr Cheng Yan Fan, Beijing, China
A*30:239	1789152	PQ466179	Prof Maria Gerbase De Lima, Sao Paulo, Brazil
A*30:240N	HG00055432	PV174109	Histogenetics, Ossining, USA
A*31:236	0123283005940	PP548295	Mrs Hanan Al Anazi, Riyadh, Saudi Arabia
A*31:237	HG00055316	PQ736462	Histogenetics, Ossining, USA
A*31:238	HG00052650	PQ241307	Histogenetics, Ossining, USA
A*31:239	2024112680	PQ872728	Dr Mingrui Huo, Beijing, China
A*32:01:01:43	24404872	PQ699975	Dr Luis A Marin, Albacete, Spain
A*32:01:64	M 8886	PV008119	Dr Thibault Pajot, Lille, France
A*32:192	1757882	PQ466175	Prof Maria Gerbase De Lima, Sao Paulo, Brazil
A*32:193	HG00055392	PQ881809	Histogenetics, Ossining, USA
A*32:194	HG00055395	PQ881812	Histogenetics, Ossining, USA
A*33:03:01:29	0124184005391	PQ682514	Mrs Hanan Al Anazi, Riyadh, Saudi Arabia
A*33:277N	HG00050294	ON987338	Histogenetics, Ossining, USA
A*33:278	BRCBC111	PQ811032	Ms DongMei Li, Beijing, China
A*33:279	HG00055399	PV009098	Histogenetics, Ossining, USA
A*33:280	MWJA33	PV031617	Dr Yuanyuan Jing, Beijing, China
A*33:281	HG00055434	PV174111	Histogenetics, Ossining, USA
A*68:01:02:37	HG00055314	PQ736460	Histogenetics, Ossining, USA
A*68:02:27	1033‐0000	PV097232	Dr Bobbie Rhodes‐Clark, Mather, USA
A*68:23:02	1790096	PQ466168	Prof Maria Gerbase De Lima, Sao Paulo, Brazil
A*68:327	HG00055354	PQ778465	Histogenetics, Ossining, USA
A*68:328	HG00055396	PQ881813	Histogenetics, Ossining, USA
A*68:329	1652085	PQ466184	Prof Maria Gerbase De Lima, Sao Paulo, Brazil
A*68:330	1702494	PQ349840	Prof Maria Gerbase De Lima, Sao Paulo, Brazil
A*68:331	G 4552	PV008121	Dr Thibault Pajot, Lille, France
A*74:01:12	1808174	PQ466181	Prof Maria Gerbase De Lima, Sao Paulo, Brazil
A*74:53	1827087	PQ349842	Prof Maria Gerbase De Lima, Sao Paulo, Brazil
B*07:05:01:08	204‐01776	PQ217972	Miss Yue Han, Xi'an, China
B*07:526N	HG00055326	PQ736472	Histogenetics, Ossining, USA
B*07:527	HG00050297	ON987406	Histogenetics, Ossining, USA
B*07:528	MC009B07var	PQ768425	Dr Zeying Du, Jacksonville, USA
B*07:529	120535	PQ932520	Dr Francisco de Paula Sanchez Gordo, Malaga, Spain
B*07:530	HG00055440	PV138031, PV138031	Histogenetics, Ossining, USA
B*07:531	KSR161931	PV219216	Dr Maria Loginova, Kirov, Russia
B*08:01:01:77	GR‐EVA‐60298	PV037133	Dr Diamanto Kouniaki, Athens, Greece
B*08:01:80	LIT2515‐2023	OR459926	Dr Mariarosa Battarra, Roma, Italy
B*08:01:81	KSR218149	PQ784001	Dr Maria Loginova, Kirov, Russia
B*08:01:82	HG00055403	PQ881831	Histogenetics, Ossining, USA
B*08:333N	KSR145087	PV219217	Dr Maria Loginova, Kirov, Russia
B*08:334	KSR111053	PV219218	Dr Maria Loginova, Kirov, Russia
B*13:02:01:32	GR‐EVA‐60373	PV076210	Dr Diamanto Kouniaki, Athens, Greece
B*13:02:45	HG00055320	PQ736466	Histogenetics, Ossining, USA
B*13:204	JH0156	PQ657625	Dr Maria Bettinotti, Baltimore, USA
B*13:205N	BRCBC222	PQ811033	Ms DongMei Li, Beijing, China
B*13:206	GXDB13	PQ890123	Ms Caifeng Long, Beijing, China
B*13:207	HN2408	PQ412958	Dr Zheng Liu, Zhengzhou, China
B*14:02:34	KSR215857	PV219219	Dr Maria Loginova, Kirov, Russia
B*15:01:01:71Q	29001895	PQ857572	Dr Daniel Fuerst, Ulm, Germany
B*15:01:92	5520	PQ600307	Dr Dan Zhang, Beijing, China
B*15:11:01:02	P17L2401292	PQ790064	Ms Wenqian Song, Dalian, China
B*15:15:03	HG00055444	PV174118	Histogenetics, Ossining, USA
B*15:21:01:03	HG00055318	PQ736464	Histogenetics, Ossining, USA
B*15:725	900612490100826	PQ720672	Prof Faming Zhu, Hangzhou, China
B*15:726	20QB012584	OZ121607	Dr Janette Kwok, Pokfulam Road, Hong Kong
B*15:727	5171723	PQ787215	Dr Antonio Balas, Madrid, Spain
B*15:728	HG00055363	PQ801769	Histogenetics, Ossining, USA
B*15:729	25200870, 25202885	PV021449, PV111836	Dr Daniel Fuerst, Ulm, Germany
B*15:730	2025TB02233	PV176822	Dr Keming Du, Shanghai, China
B*18:05:01:03	933224	PV083173	Dr Vasiliki Kitsiou, Athens, Greece
B*18:255	1803471	PQ466182	Prof Maria Gerbase De Lima, Sao Paulo, Brazil
B*18:256	HG00055439	PV094885	Histogenetics, Ossining, USA
B*27:05:64	2410858809	PQ860820	Dr Jonathan Visentin, Bordeaux, France
B*27:288	KSR162306	PQ784002	Dr Maria Loginova, Kirov, Russia
B*27:289	KSR126334	PQ784003	Dr Maria Loginova, Kirov, Russia
B*27:290	2172106	PQ787214	Dr Antonio Balas, Madrid, Spain
B*27:291	42342061	PQ817302	Dr Romain Ferru‐Clement, Poitiers, France
B*27:292	20QB012759	OZ121608	Dr Janette Kwok, Pokfulam Road, Hong Kong
B*27:293	24227999	PQ932027	Dr Daniel Fuerst, Ulm, Germany
B*27:294	KSR190595	PV219220	Dr Maria Loginova, Kirov, Russia
B*35:01:83	HG00055361	PQ801767	Histogenetics, Ossining, USA
B*35:03:45	KSR206054	PQ784005	Dr Maria Loginova, Kirov, Russia
B*35:08:01:14	24405025	PV015026	Dr Luis A Marin, Albacete, Spain
B*35:514:02	42450113	PQ817303	Dr Romain Ferru‐Clement, Poitiers, France
B*35:637	KSR201332	PQ784004	Dr Maria Loginova, Kirov, Russia
B*35:638	BRCBCY003	PQ816950	Dr Yu Jie Wen, Beijing, China
B*35:639	RP‐292622	PQ827118	Dr Neifi Hassan Saloum Deghaide, Ribeirao Preto, Brazil
B*35:640	10282402337	PV050369	Dr Nedal Taha, Dedham, USA
B*35:641	KSR162520	PV219221	Dr Maria Loginova, Kirov, Russia
B*37:01:35	HG00055365	PQ820456	Histogenetics, Ossining, USA
B*37:01:36	HG00055443	PV174117	Histogenetics, Ossining, USA
B*38:123	2206901	PQ569834	Dr Mirzokhid Rakhmanov, Freiburg, Germany
B*38:124	5712401035	PV031721	Prof Faming Zhu, Hangzhou, China
B*38:125	MC010B38v	PV076227	Dr Zeying Du, Jacksonville, USA
B*39:01:01:31	332524	PV083171	Dr Vasiliki Kitsiou, Athens, Greece
B*39:229	KSR162273	PQ784006	Dr Maria Loginova, Kirov, Russia
B*39:230	HG00055438	PV094884	Histogenetics, Ossining, USA
B*40:01:93	900612490103026	PQ720673	Prof Faming Zhu, Hangzhou, China
B*40:01:94	20QB012966	OZ121609	Dr Janette Kwok, Pokfulam Road, Hong Kong
B*40:160:01:02	50100119810	PV035082	Dr Prabin Kumar, Ahmedabad, India
B*40:596:02	017398711	PV157335	Mrs Ornella Senoussi, La Tronche, France
B*40:603	HG00055319	PQ736465	Histogenetics, Ossining, USA
B*40:604	HG00055359	PQ778463	Histogenetics, Ossining, USA
B*40:605	2024TB23076	PQ850025	Dr Keming Du, Shanghai, China
B*40:606N	JH0161	PV170733	Dr Maria Bettinotti, Baltimore, USA
B*44:02:01:88	24405002	PQ998755	Dr Luis A Marin, Albacete, Spain
B*44:03:01:76	24404843	PQ660765	Dr Luis A Marin, Albacete, Spain
B*44:03:01:77	202320963	PQ305756	Dr Amalia Tejeda Velarde, Salamanca, Spain
B*44:03:73	HG00055322	PQ736468	Histogenetics, Ossining, USA
B*44:03:74	25529380	PQ998991	Mr Abootaleb Rahmanian, Portland, USA
B*44:03:75	HG00055441	PV138032	Histogenetics, Ossining, USA
B*44:357:02	2411029094	PQ860821	Dr Jonathan Visentin, Bordeaux, France
B*44:425	015068	PQ723759	Dr Donatella Londero, Udine, Italy
B*44:426	KSR218223	PQ784007	Dr Maria Loginova, Kirov, Russia
B*44:427Q	HG00055366	PQ820457	Histogenetics, Ossining, USA
B*44:428	HG00055362	PQ801768	Histogenetics, Ossining, USA
B*44:429N	UBH0013	PQ586428	Mr Marc Kleiser, Basel, Switzerland
B*44:430	KSR209172	PV219223	Dr Maria Loginova, Kirov, Russia
B*44:444:02	HG00055321	PQ736467	Histogenetics, Ossining, USA
B*46:104	BRCBCY002	PQ816949	Dr Yu Jie Wen, Beijing, China
B*46:105	HN2407	PQ412956	Dr Zheng Liu, Zhengzhou, China
B*46:106	BRCBC10056	PV165926	Prof Jie Wang, Beijing, China
B*47:14	HG00055325	PQ736471	Histogenetics, Ossining, USA
B*48:01:01:15	P19L2401324	PQ790065	Ms Wenqian Song, Dalian, China
B*48:05:02	HG00055437	PV094883, PV094883	Histogenetics, Ossining, USA
B*49:89N	KSR129687	PQ784008	Dr Maria Loginova, Kirov, Russia
B*49:90	KSR129622	PQ784009	Dr Maria Loginova, Kirov, Russia
B*50:29	1818284	PQ466183	Prof Maria Gerbase De Lima, Sao Paulo, Brazil
B*51:01:01:126	GR‐EVA‐58959	PV076211	Dr Diamanto Kouniaki, Athens, Greece
B*51:17:02	KSR132293	PQ784010	Dr Maria Loginova, Kirov, Russia
B*52:30:02	HG00055436	PV094882	Histogenetics, Ossining, USA
B*53:81N	HG00055435	PV094881	Histogenetics, Ossining, USA
B*55:02:01:10	201‐00561	PQ217978	Miss Yue Han, Xi'an, China
B*56:01:01:21	HG00055317	PQ736463	Histogenetics, Ossining, USA
B*57:03:10	120757	PV098985	Dr Francisco de Paula Sanchez Gordo, Malaga, Spain
B*57:192	HG00055364, HG00055323, HG00055324	PQ736469, PQ736470, PQ801770	Histogenetics, Ossining, USA
B*57:193	1813587	PQ382545	Prof Maria Gerbase De Lima, Sao Paulo, Brazil
B*57:194	HG00055404	PV037253	Histogenetics, Ossining, USA
C*01:02:96	21QB001787	OZ121610	Dr Janette Kwok, Pokfulam Road, Hong Kong
C*01:285Q	AMC‐E‐010	PQ452786	Mrs So‐Jung Choi, Seoul, South Korea
C*01:286N	HG00055371	PQ801762	Histogenetics, Ossining, USA
C*01:287	KSr191954	PQ845327	Dr Maria Loginova, Kirov, Russia
C*01:288	20QB000177	MZ288722	Dr Janette Kwok, Pokfulam Road, Hong Kong
C*02:31:02	KSR210274	PQ845328	Dr Maria Loginova, Kirov, Russia
C*03:02:36	21QB002466	OZ121611	Dr Janette Kwok, Pokfulam Road, Hong Kong
C*03:03:01:72	P4L2401039	PQ202042	Ms Wenqian Song, Dalian, China
C*03:04:115	HG00055370	PQ778473	Histogenetics, Ossining, USA
C*03:04:116	HG00055405	PQ881817	Histogenetics, Ossining, USA
C*03:180:02	1732686	PQ466177	Prof Maria Gerbase De Lima, Sao Paulo, Brazil
C*03:642:02	202408519	PQ305755	Dr Amalia Tejeda Velarde, Salamanca, Spain
C*03:704	HG00055330	PQ736479	Histogenetics, Ossining, USA
C*03:705	HG00055331	PQ736480	Histogenetics, Ossining, USA
C*03:706N	HG00031856	OM688053	Histogenetics, Ossining, USA
C*03:707	7670HE	PQ816215	Dr Dan Zhang, Beijing, China
C*03:708	1784633	PQ466173	Prof Maria Gerbase De Lima, Sao Paulo, Brazil
C*03:709N	1797448	PQ324632	Prof Maria Gerbase De Lima, Sao Paulo, Brazil
C*03:710	KSR218527	PQ845331	Dr Maria Loginova, Kirov, Russia
C*03:711	BRCBCY004	PQ900557	Dr Yu Jie Wen, Beijing, China
C*03:712	BRCBC6323	PQ811586	Prof Jie Wang, Beijing, China
C*03:713	JH0162	PV170734	Dr Maria Bettinotti, Baltimore, USA
C*04:01:01:195	GR‐EVA‐55103	PV092590	Dr Diamanto Kouniaki, Athens, Greece
C*04:03:12	HG00055410	PQ881822	Histogenetics, Ossining, USA
C*04:540	HG00051961	PP471056	Histogenetics, Ossining, USA
C*04:541	SIMBH361666	PQ768513	Dr Renata Monti Rocha, Belo Horizonte, Brazil
C*04:542N	BRCBC555	PQ811036	Ms DongMei Li, Beijing, China
C*04:543	KSR113832	PQ845332	Dr Maria Loginova, Kirov, Russia
C*04:544	HG00055412	PQ881824	Histogenetics, Ossining, USA
C*04:545	HG00055409	PQ881821	Histogenetics, Ossining, USA
C*04:546	HG00055415	PV037246	Histogenetics, Ossining, USA
C*04:547	KFSH‐D_2403061930	PQ800092	Dr Rabab AlAttas, Dammam, Saudi Arabia
C*05:01:01:94	ligh041224	OK095308	Mr Alessandro Pirri, Curitiba, Brazil
C*06:02:01:104Q	24226828	PQ678884	Dr Daniel Fuerst, Ulm, Germany
C*06:02:01:105	P10L2401179	PQ827546	Ms Wenqian Song, Dalian, China
C*06:02:121	TTI5561	PV053415	Dr Cathi Murphey, San Antonio, USA
C*06:400Q	HG00055276	PQ594964	Histogenetics, Ossining, USA
C*06:401Q	BRCBC4826	PQ816217	Dr Dan Zhang, Beijing, China
C*07:01:01:145	HG00055329	PQ736478	Histogenetics, Ossining, USA
C*07:01:01:146	HG00055368	PQ778471	Histogenetics, Ossining, USA
C*07:01:02:17	HG00055369	PQ778472	Histogenetics, Ossining, USA
C*07:01:129	HG00055408	PQ881820	Histogenetics, Ossining, USA
C*07:02:163	HG00055334	PQ736483	Histogenetics, Ossining, USA
C*07:02:164	322089	PQ817130	Dr Raquel Fabreti, Belo Horizonte, Brazil
C*07:640:02	2024TB20789	PQ784214	Dr Keming Du, Shanghai, China
C*07:748:02	HG00055333	PQ736482	Histogenetics, Ossining, USA
C*07:1174	562851	PQ675320	Mrs Tamara Passos, Salvador, Brazil
C*07:1175	1043538395	OZ206346	Dr Thomas Goeury, Geneva, Switzerland
C*07:1176	HG00055336	PQ736485	Histogenetics, Ossining, USA
C*07:1177	HG00055335	PQ736484	Histogenetics, Ossining, USA
C*07:1178	015065	PQ723758	Dr Donatella Londero, Udine, Italy
C*07:1179	23N00266	PQ768536	Mrs Yanbo Nie, Tianjin, China
C*07:1180	480066	PQ586447	Prof Edward KL Yang, Hualien, Taiwan
C*07:1181	BRCBC666	PQ655383	Ms DongMei Li, Beijing, China
C*07:1182	HG00055407	PQ881819	Histogenetics, Ossining, USA
C*07:1183	UERJ‐C21384	PV091010	Dr Romulo Vianna, Rio de Janeiro, Brazil
C*07:1184	2411093807	PV067294	Dr Jonathan Visentin, Bordeaux, France
C*07:1185	2024112674	PQ872729	Dr Mingrui Huo, Beijing, China
C*07:1186	HG00055447	PV174115	Histogenetics, Ossining, USA
C*07:1187	2404200	PQ963287	Dr Paul Rouzaire, Clermont‐Ferrand, France
C*08:22:01:03	A9L2401476	PQ827547	Ms Wenqian Song, Dalian, China
C*08:301	2024243	PQ821893	Ms Muyesar Yisimayili, Urumqi, China
C*08:302N	1798823	PQ324631	Prof Maria Gerbase De Lima, Sao Paulo, Brazil
C*08:303N	1827090	PQ382549	Prof Maria Gerbase De Lima, Sao Paulo, Brazil
C*08:304	KSR153500	PQ845333	Dr Maria Loginova, Kirov, Russia
C*08:305Q	LYSC08	PV016607	Dr Yuanyuan Jing, Beijing, China
C*12:03:94	HG00055373	PQ801764	Histogenetics, Ossining, USA
C*12:03:95	KSR159436	PQ845334	Dr Maria Loginova, Kirov, Russia
C*12:03:96	KSR104231	PQ845335	Dr Maria Loginova, Kirov, Russia
C*12:431	NOS_LOU	PV165473	Mr Arnaud Daly, Strasbourg, France
C*12:432N	HG00055445	PV094891	Histogenetics, Ossining, USA
C*12:433	KSR111741	PV219224	Dr Maria Loginova, Kirov, Russia
C*14:169	2024TB14635	PQ340685	Dr Keming Du, Shanghai, China
C*14:170	HG00055446	PV174114	Histogenetics, Ossining, USA
C*15:02:67	HG00055332	PQ736481	Histogenetics, Ossining, USA
C*15:292	2023200	PQ821894	Mr Weipeng Guo, Urumqi, China
C*15:293	HG00055411	PQ881823	Histogenetics, Ossining, USA
C*16:01:01:43	HG00055328	PQ736477	Histogenetics, Ossining, USA
C*16:226	1789036	PQ382546	Prof Maria Gerbase De Lima, Sao Paulo, Brazil
C*16:227	RP‐293010	PQ999095	Dr Neifi Hassan Saloum Deghaide, Ribeirao Preto, Brazil
C*16:228	KSR127481	PQ845336	Dr Maria Loginova, Kirov, Russia
C*16:229N	HG00055414	PV037245	Histogenetics, Ossining, USA
C*16:230	25202492	PV075034	Dr Daniel Fuerst, Ulm, Germany
C*17:03:01:11	HG00055327	PQ736476	Histogenetics, Ossining, USA
C*17:03:01:12	HG00055367	PQ778470	Histogenetics, Ossining, USA
C*17:03:06	KSR187834	PQ845337	Dr Maria Loginova, Kirov, Russia
E*01:01:46	P15775‐1	PV031725	Prof Faming Zhu, Hangzhou, China
E*01:152	NL‐Z83	PQ587575	Dr Nitalia Naidoo, Durban, South Africa
F*01:01:02:17	A6L2401442	PQ827548	Ms Wenqian Song, Dalian, China
G*01:01:01:39	P15L2401244	PQ827549	Ms Wenqian Song, Dalian, China
DRA*01:01:08	1048334403	OZ206351	Dr Thomas Goeury, Geneva, Switzerland
DRA*01:18	4012280831	OZ206349	Dr Thomas Goeury, Geneva, Switzerland
DRB1*01:156	KSR192302	PV219225	Dr Maria Loginova, Kirov, Russia
DRB1*03:01:01:28	0823‐39583	PV087383	Dr Yanping Huang, Philadelphia, USA
DRB1*03:219	KSR183207	PQ676736	Dr Maria Loginova, Kirov, Russia
DRB1*03:220	HG00055348	PQ736489	Histogenetics, Ossining, USA
DRB1*03:221	031924CA	PV022083	Dr Nedal Taha, Dedham, USA
DRB1*03:222	HG00055460	PV174112	Histogenetics, Ossining, USA
DRB1*04:171:02	KSR159994	PQ676737	Dr Maria Loginova, Kirov, Russia
DRB1*04:392	KSR118465	PQ676733	Dr Maria Loginova, Kirov, Russia
DRB1*04:393	HG00055347	PQ736488	Histogenetics, Ossining, USA
DRB1*04:394	HG00055346	PQ736487	Histogenetics, Ossining, USA
DRB1*04:395	2410888899	PQ860818	Dr Jonathan Visentin, Bordeaux, France
DRB1*04:396	HG00055429	PQ881832	Histogenetics, Ossining, USA
DRB1*04:397	HG00055430	PV037247	Histogenetics, Ossining, USA
DRB1*04:398	HG00055456	PV094886	Histogenetics, Ossining, USA
DRB1*07:168	KSR190637	PQ676738	Dr Maria Loginova, Kirov, Russia
DRB1*07:169	HG00055386	PQ801773	Histogenetics, Ossining, USA
DRB1*08:135	65240246921	PQ268861	Mr Gerald Bertrand, Rennes Cedex, France
DRB1*08:136	H240550	PQ720674	Prof Faming Zhu, Hangzhou, China
DRB1*11:01:51	42439318	PQ588446	Dr Romain Ferru‐Clement, Poitiers, France
DRB1*11:01:52	KSR128458	PQ676740	Dr Maria Loginova, Kirov, Russia
DRB1*11:02:08	RP‐288808	PQ879617	Dr Neifi Hassan Saloum Deghaide, Ribeirao Preto, Brazil
DRB1*11:341	6185869‐JPV	PQ473687	Dr Jeane Visentainer, Maringa, Brazil
DRB1*11:342	KSR190727	PQ676741	Dr Maria Loginova, Kirov, Russia
DRB1*11:343	2406712	PQ660070	Dr Mirzokhid Rakhmanov, Freiburg, Germany
DRB1*11:344	2024TB14310	PQ340684	Dr Keming Du, Shanghai, China
DRB1*11:345	HG00055457	PV094887	Histogenetics, Ossining, USA
DRB1*11:346	HG00055458	PV094888	Histogenetics, Ossining, USA
DRB1*12:115	900612490095826	PQ720675	Prof Faming Zhu, Hangzhou, China
DRB1*12:116	5712402961	PQ720676	Prof Faming Zhu, Hangzhou, China
DRB1*13:368	KSR98863	PQ676742	Dr Maria Loginova, Kirov, Russia
DRB1*13:369	MC30450015	PV176856	Dr Beatrice Pedron, Paris, France
DRB1*14:274	HG00055351	PQ736492	Histogenetics, Ossining, USA
DRB1*14:275	JAU‐36286	PQ963010	Mr Marcos Paulo Vicari Souza, Jau, Brazil
DRB1*15:03:06	315910	PQ882775	Dr Raquel Fabreti, Belo Horizonte, Brazil
DRB1*15:236N	HG00055349	PQ736490	Histogenetics, Ossining, USA
DRB1*15:237	5712401473	PV031723	Prof Faming Zhu, Hangzhou, China
DRB1*16:80	2024TB11580	PQ304861	Dr Keming Du, Shanghai, China
DRB1*16:81	52500324	PQ998982	Dr Andreas Heinold, Essen, Germany
DRB3*01:01:02:07	PSCDA0095	PV177808	Dr Seik‐Soon Khor, Tokyo, Japan
DRB3*01:01:02:08	PSCDA0440	PV177809	Dr Seik‐Soon Khor, Tokyo, Japan
DRB3*01:134	201‐00436	PV224481	Ms Yuanli Zhao, Xi'an, China
DRB3*01:135	204_00608	PV224483	Ms Yuanli Zhao, Xi'an, China
DRB3*01:136	204‐01601	PV224487	Ms Yuanli Zhao, Xi'an, China
DRB3*01:137	204‐01828	PV224488	Ms Yuanli Zhao, Xi'an, China
DRB3*01:138:01:01	201‐00541	PV224477	Ms Yuanli Zhao, Xi'an, China
DRB3*01:138:01:02	201‐00559	PV224478	Ms Yuanli Zhao, Xi'an, China
DRB3*02:02:01:19	201_01324	PQ725993	Ms Yuanli Zhao, Xi'an, China
DRB3*02:02:01:20	204_00707	PQ725989	Ms Yuanli Zhao, Xi'an, China
DRB3*02:02:01:21	201‐00460	PQ684255	Ms Yuanli Zhao, Xi'an, China
DRB3*02:02:01:22	201‐00561	PQ634720	Ms Yuanli Zhao, Xi'an, China
DRB3*02:02:01:23	201‐00329	PQ634721	Ms Yuanli Zhao, Xi'an, China
DRB3*02:02:01:24	201_01225	PQ634722	Ms Yuanli Zhao, Xi'an, China
DRB3*02:02:01:25	204_00431	PV158726	Ms Yuanli Zhao, Xi'an, China
DRB3*02:216	25201102	PV021447	Dr Daniel Fuerst, Ulm, Germany
DRB3*03:01:17:01	201_00844	PQ862238	Ms Yuanli Zhao, Xi'an, China
DRB3*03:01:17:02	204‐01726	PQ862243	Ms Yuanli Zhao, Xi'an, China
DRB3*03:01:18	201_00625S	PV158724	Ms Yuanli Zhao, Xi'an, China
DRB3*03:79:01	204_00364	PQ862240	Ms Yuanli Zhao, Xi'an, China
DRB3*03:79:02	204‐01770S	PQ862241	Ms Yuanli Zhao, Xi'an, China
DRB3*03:80:01:01	201_01403	PQ862239	Ms Yuanli Zhao, Xi'an, China
DRB3*03:80:01:02	1292SS	PQ862242	Ms Yuanli Zhao, Xi'an, China
DRB4*01:01:01:05	204‐01770	PP955966	Ms Yuanli Zhao, Xi'an, China
DRB4*01:01:01:06	201‐00009S	PQ809490	Ms Yuanli Zhao, Xi'an, China
DRB4*01:01:14	JH0155	PQ653880	Dr Maria Bettinotti, Baltimore, USA
DRB4*01:02:01:02	201‐00414	PP955976	Ms Yuanli Zhao, Xi'an, China
DRB4*01:03:01:27	201‐00329	PP955963	Ms Yuanli Zhao, Xi'an, China
DRB4*01:03:01:28N	201‐00564	PQ539412	Ms Yuanli Zhao, Xi'an, China
DRB4*01:03:01:29	201_00920	PQ539413	Ms Yuanli Zhao, Xi'an, China
DRB4*01:03:01:30	201‐00510	PQ809489	Ms Yuanli Zhao, Xi'an, China
DRB4*01:03:02:04	201‐00389	PP955968	Ms Yuanli Zhao, Xi'an, China
DRB4*01:03:02:05	201‐00506	PP955969	Ms Yuanli Zhao, Xi'an, China
DRB4*01:03:02:06	204‐00789, 201–00605S	PP955972	Ms Yuanli Zhao, Xi'an, China
DRB4*01:03:02:07	201_00711	PP955973	Ms Yuanli Zhao, Xi'an, China
DRB4*01:03:02:08	204‐00807	PQ539416	Ms Yuanli Zhao, Xi'an, China
DRB4*01:03:02:09	204‐01744	PP955974	Ms Yuanli Zhao, Xi'an, China
DRB4*01:03:02:10	204‐01776	PP955975	Ms Yuanli Zhao, Xi'an, China
DRB4*01:03:37	JH0154	PQ653879	Dr Maria Bettinotti, Baltimore, USA
DRB4*01:191	d14208	PV165529	Dr Cathi Murphey, San Antonio, USA
DRB4*01:192N	HG00055463	PV094890	Histogenetics, Ossining, USA
DRB4*01:193	2404500	PQ963286	Dr Paul Rouzaire, Clermont‐Ferrand, France
DRB5*01:01:01:12	22WB00210	PQ559725	Ms Yuanli Zhao, Xi'an, China
DRB5*01:01:01:13	201‐00343	PQ476165	Ms Yuanli Zhao, Xi'an, China
DRB5*01:02:01:02	201‐00176	PQ772348	Ms Yuanli Zhao, Xi'an, China
DRB5*01:02:01:03	204_00599	PQ772349	Ms Yuanli Zhao, Xi'an, China
DRB5*01:144N	204‐01943	PQ809488	Ms Yuanli Zhao, Xi'an, China
DRB5*01:145N	201_01390	PQ809487	Ms Yuanli Zhao, Xi'an, China
DQA1*01:01:15	MRB2743	PP763747	Dr Eugene Albert, Dolgoprudny, Russian Federation
DQA1*01:01:16	408014086	PQ811904	Mr Jobson Nascimento, Recife, Brazil
DQA1*01:02:01:33	P14L2401265	PQ824405	Ms Wenqian Song, Dalian, China
DQA1*01:02:01:34	P22L2401425	PQ824408	Ms Wenqian Song, Dalian, China
DQA1*01:02:02:07	P8L2401143	PQ824404	Ms Wenqian Song, Dalian, China
DQA1*01:03:01:18	24405270	PV207324	Dr Luis A Marin, Albacete, Spain
DQA1*01:100:02	508401	PQ438153	Mr Alessandro Pirri, Curitiba, Brazil
DQA1*01:180	HG00055423	PQ881830	Histogenetics, Ossining, USA
DQA1*02:50	HG00055424	PV037252	Histogenetics, Ossining, USA
DQA1*05:01:21	MC011DQA105v	PV092595	Dr Zeying Du, Jacksonville, USA
DQA1*05:05:01:44	P8L2401143	PQ824403	Ms Wenqian Song, Dalian, China
DQA1*05:05:01:45	P20L2401351	PQ824407	Ms Wenqian Song, Dalian, China
DQA1*05:05:24	Amiens16	PQ699790	Dr Nicolas Guillaume, Amiens, France
DQA1*05:118:01:02	202417361	PQ855750	Dr Amalia Tejeda Velarde, Salamanca, Spain
DQA1*05:121	015241	PQ723762	Dr Donatella Londero, Udine, Italy
DQA1*05:122	202403477	PQ305754	Dr Amalia Tejeda Velarde, Salamanca, Spain
DQA1*05:123	120394	PQ789260	Dr Francisco de Paula Sanchez Gordo, Malaga, Spain
DQA1*06:01:01:06	P14L2401265	PQ824406	Ms Wenqian Song, Dalian, China
DQB1*05:01:01:41	24818997	PV241190	Dr Luis A Marin, Albacete, Spain
DQB1*05:01:55	0423‐34003	PP336907	Dr Yanping Huang, Philadelphia, USA
DQB1*05:03:39	KSR150488	PQ665508	Dr Maria Loginova, Kirov, Russia
DQB1*05:358	2024TB11075	PQ304863	Dr Keming Du, Shanghai, China
DQB1*06:01:01:10	UERJ‐C20854	PV091012	Dr Romulo Vianna, Rio de Janeiro, Brazil
DQB1*06:02:01:43	P22L2401425	PQ800100	Ms Wenqian Song, Dalian, China
DQB1*06:02:66	DKMS‐LSL_ID20720832	OZ210315	DKMS Life Sciences Lab., Dresden, Germany
DQB1*06:03:54	DKMS‐LSL_ID21387962	OZ210320	DKMS Life Sciences Lab., Dresden, Germany
DQB1*06:03:54	KSR109119	PQ665509	Dr Maria Loginova, Kirov, Russia
DQB1*06:03:55	KSR173436	PV219229	Dr Maria Loginova, Kirov, Russia
DQB1*06:04:01:11	2935424	PV083175	Dr Vasiliki Kitsiou, Athens, Greece
DQB1*06:273:02	5712401420	PQ720677	Prof Faming Zhu, Hangzhou, China
DQB1*06:526	KSR171174	PQ665510	Dr Maria Loginova, Kirov, Russia
DQB1*06:527	DKMS‐LSL_ID20960076	OZ210317	DKMS Life Sciences Lab., Dresden, Germany
DQB1*06:528	2024TB16155	PQ394579	Dr Keming Du, Shanghai, China
DQB1*06:529	24405032	PV015025	Dr Luis A Marin, Albacete, Spain
DQB1*06:530	021982473	PQ660071	Dr Xavier Fournel, Saint Etienne, France
DQB1*06:531	KFSH‐D_2406042261	PQ800091	Dr Rabab AlAttas, Dammam, Saudi Arabia
DQB1*02:01:51	KSR182742	PQ665503	Dr Maria Loginova, Kirov, Russia
DQB1*02:02:32	1173242	PQ872708	Dr Antonio Balas, Madrid, Spain
DQB1*02:246	DKMS‐LSL_ID21145259	OZ210318	DKMS Life Sciences Lab., Dresden, Germany
DQB1*02:247	DKMS‐LSL_ID21421050	OZ210322	DKMS Life Sciences Lab., Dresden, Germany
DQB1*02:248	DKMS‐LSL_ID21332048, DKMS‐LSL_ID20600494	OZ210312, OZ210314	DKMS Life Sciences Lab., Dresden, Germany
DQB1*02:249	DKMS‐LSL_ID20914229	OZ210311	DKMS Life Sciences Lab., Dresden, Germany
DQB1*02:250	DKMS‐LSL_ID20782180	OZ210310	DKMS Life Sciences Lab., Dresden, Germany
DQB1*02:251N	1173290	PQ872709	Dr Antonio Balas, Madrid, Spain
DQB1*03:01:01:74	DKMS‐LSL_ID21353448, DKMS‐LSL_ID20861086	OZ210316, OZ210319	DKMS Life Sciences Lab., Dresden, Germany
DQB1*03:01:01:75	58839‐110024	PV083177	Dr Vasiliki Kitsiou, Athens, Greece
DQB1*03:01:01:76	319424	PV083174	Dr Vasiliki Kitsiou, Athens, Greece
DQB1*03:01:01:77	24405161	PV129946	Dr Luis A Marin, Albacete, Spain
DQB1*03:01:69	316683	PQ882776	Dr Raquel Fabreti, Belo Horizonte, Brazil
DQB1*03:02:48	KSR102371	PV219228	Dr Maria Loginova, Kirov, Russia
DQB1*03:19:01:03	713424	PV083172	Dr Vasiliki Kitsiou, Athens, Greece
DQB1*03:390:02	KSR113742	PQ665504	Dr Maria Loginova, Kirov, Russia
DQB1*03:573	KSR125100	PQ665505	Dr Maria Loginova, Kirov, Russia
DQB1*03:574	HR5365473	PQ687603	Prof Renata Zunec, Zagreb, Croatia
DQB1*03:575	LHSC001	PQ660887	Dr Abubaker ME Sidahmed, London, Canada
DQB1*03:576Q	DKMS‐LSL_ID21351670	OZ210313	DKMS Life Sciences Lab., Dresden, Germany
DQB1*03:577	2926‐2024	PQ855745	Dr Mariarosa Battarra, Roma, Italy
DQB1*03:578N	5712401121	PQ800079	Prof Faming Zhu, Hangzhou, China
DQB1*03:579	KSR217148	PV219227	Dr Maria Loginova, Kirov, Russia
DQB1*04:01:09	P10L2401179	PQ667744	Ms Wenqian Song, Dalian, China
DQB1*04:105	KSR160027, KSR204216	PQ665506	Dr Maria Loginova, Kirov, Russia
DQB1*04:106	BRCBCY001	PQ816948	Dr Yu Jie Wen, Beijing, China
DQB1*04:107	6240308_NTB	PQ963842	Dr Jeane Visentainer, Maringa, Brazil
DQB1*04:108	202419854	PV112449	Dr Amalia Tejeda Velarde, Salamanca, Spain
DPA1*01:03:01:97	24819789	PQ505254	Dr Luis A Marin, Albacete, Spain
DPA1*01:03:01:98	24405106	PV037737	Dr Luis A Marin, Albacete, Spain
DPA1*01:03:01:99	925424	PV083178	Dr Vasiliki Kitsiou, Athens, Greece
DPA1*01:232	120520	PQ932519	Dr Francisco de Paula Sanchez Gordo, Malaga, Spain
DPA1*02:01:36	RP‐288195	PQ603016	Dr Neifi Hassan Saloum Deghaide, Ribeirao Preto, Brazil
DPA1*02:01:37	61036541	PQ858688	Dr Dolores Planelles, Valencia, Spain
DPA1*02:01:38	BMD8719	PQ868297	Dr Cathi Murphey, San Antonio, USA
DPA1*02:02:02:22	1416924	PV083179	Dr Vasiliki Kitsiou, Athens, Greece
DPA1*02:07:05	HG00055374	PQ801760	Histogenetics, Ossining, USA
DPA1*02:136:02	0922‐24989	PV087382	Dr Yanping Huang, Philadelphia, USA
DPA1*02:150	120134	PQ657865	Dr Francisco de Paula Sanchez Gordo, Malaga, Spain
DPA1*02:151	202420816	PQ855793	Dr Amalia Tejeda Velarde, Salamanca, Spain
DPA1*02:152	120357	PQ858692	Dr Francisco de Paula Sanchez Gordo, Malaga, Spain
DPA1*04:08	24229T0006	PQ595900	Ms Sarah Peacock, Cambridge, United Kingdom
DPB1*01:01:17	DKMS‐LSL_ID21574769	OZ211631	DKMS Life Sciences Lab., Dresden, Germany
DPB1*01:01:18	DKMS‐LSL_ID21169267	OZ211636	DKMS Life Sciences Lab., Dresden, Germany
DPB1*02:01:02:109	A11L2401493	PQ784966	Ms Wenqian Song, Dalian, China
DPB1*02:01:02:110	204_00707	PQ824435	Miss Yue Han, Xi'an, China
DPB1*02:01:02:111	P14L2401265	PQ824400	Ms Wenqian Song, Dalian, China
DPB1*02:01:02:112	A5L2401420	PQ824402	Ms Wenqian Song, Dalian, China
DPB1*03:01:34	HG00055339	PQ736473	Histogenetics, Ossining, USA
DPB1*04:01:01:163	201‐00595	PQ679033	Miss Yue Han, Xi'an, China
DPB1*04:01:01:164	204‐00807	PQ824436	Miss Yue Han, Xi'an, China
DPB1*04:01:95	315089	PQ882777	Dr Raquel Fabreti, Belo Horizonte, Brazil
DPB1*04:01:96	120705	PV098984	Dr Francisco de Paula Sanchez Gordo, Malaga, Spain
DPB1*04:02:01:46	201_01290_1	PQ807226	Miss Yue Han, Xi'an, China
DPB1*04:02:33	DKMS‐LSL_ID21432010	OZ211630	DKMS Life Sciences Lab., Dresden, Germany
DPB1*05:01:01:41	201‐00326	PQ663256	Miss Yue Han, Xi'an, China
DPB1*05:01:01:42	201‐00380	PQ663259	Miss Yue Han, Xi'an, China
DPB1*05:01:01:43	201‐00606	PQ679034	Miss Yue Han, Xi'an, China
DPB1*05:01:01:44	201_00646	PQ679035	Miss Yue Han, Xi'an, China
DPB1*05:01:01:45	201_00670	PQ679036	Miss Yue Han, Xi'an, China
DPB1*05:01:01:46	201_01290_2	PQ807225	Miss Yue Han, Xi'an, China
DPB1*05:01:01:47	204_00679	PQ824434	Miss Yue Han, Xi'an, China
DPB1*05:01:01:48	P14L2401265	PQ824390	Ms Wenqian Song, Dalian, China
DPB1*05:01:01:49	P19L2401324	PQ824401	Ms Wenqian Song, Dalian, China
DPB1*05:01:25	5712401844	PQ720678	Prof Faming Zhu, Hangzhou, China
DPB1*09:01:01:03	204_00651_1	PQ824432	Miss Yue Han, Xi'an, China
DPB1*13:01:01:19	DKMS‐LSL_ID20834681	OZ211627	DKMS Life Sciences Lab., Dresden, Germany
DPB1*14:01:01:14	201_00824	PQ807224	Miss Yue Han, Xi'an, China
DPB1*14:01:01:15	204_00651	PQ807228	Miss Yue Han, Xi'an, China
DPB1*17:01:01:11	204_00669	PQ824433	Miss Yue Han, Xi'an, China
DPB1*29:01:02	175827052	PQ563177	Dr Pascal Pedini, Marseille, France
DPB1*107:01:01:02	A9L2401476	PQ784965	Ms Wenqian Song, Dalian, China
DPB1*135:01:01:03	204_00157	PQ807227	Miss Yue Han, Xi'an, China
DPB1*389:01:02	DKMS‐LSL_ID21270722	OZ211623	DKMS Life Sciences Lab., Dresden, Germany
DPB1*849:01:02	DKMS‐LSL_ID20969509	OZ211635	DKMS Life Sciences Lab., Dresden, Germany
DPB1*1677:01:01:02N	DKMS‐LSL_ID20834681	OZ211626	DKMS Life Sciences Lab., Dresden, Germany
DPB1*1758:01	202407020001	PQ726439	Mr Bin Chen, Tianjin, China
DPB1*1759:01	EUMC003	LC848300	Dr Soo‐Kyung Kim, Seoul, Republic of Korea
DPB1*1760:01	HG00050060, HG00055353	MW595458, MW595459	Histogenetics, Ossining, USA
DPB1*1761:01	HG00021525	MK616639	Histogenetics, Ossining, USA
DPB1*1762:01	17855733	PQ738197	Dr Antonio Balas, Madrid, Spain
DPB1*1763:01	SZ‐300	PQ801476	Dr Jie Liu, Shenzhen, Guangdong, China
DPB1*1764:01	DKMS‐LSL_ID21462386	OZ211624	DKMS Life Sciences Lab., Dresden, Germany
DPB1*1765:01:01:01	DKMS‐LSL_ID21193440	OZ211622	DKMS Life Sciences Lab., Dresden, Germany
DPB1*1765:01:01:02	DKMS‐LSL_ID20719633	OZ211616	DKMS Life Sciences Lab., Dresden, Germany
DPB1*1766:01Q	DKMS‐LSL_ID21092013	OZ211619	DKMS Life Sciences Lab., Dresden, Germany
DPB1*1767:01	DKMS‐LSL_ID21306697	OZ211620	DKMS Life Sciences Lab., Dresden, Germany
DPB1*1768:01	DKMS‐LSL_ID20664627	OZ211614	DKMS Life Sciences Lab., Dresden, Germany
DPB1*1769:01	DKMS‐LSL_ID21650422	OZ211634	DKMS Life Sciences Lab., Dresden, Germany
DPB1*1770:01	DKMS‐LSL_ID21585258	OZ211632	DKMS Life Sciences Lab., Dresden, Germany
DPB1*1771:01	DKMS‐LSL_ID21613901	OZ211625	DKMS Life Sciences Lab., Dresden, Germany
DPB1*1772:01	2024TB12965	PQ340682	Dr Keming Du, Shanghai, China
DPB1*1773:01	2024TB13268	OP617203	Dr Keming Du, Shanghai, China
DPB1*1774:01	2024TB20367	PQ784216	Dr Keming Du, Shanghai, China
DPB1*1775:01N	HS26345	PQ855752	Dr Cathi Murphey, San Antonio, USA
DPB1*1776:01	1172518	PQ872706	Dr Antonio Balas, Madrid, Spain
DPB1*1777:01	120515	PQ932518	Dr Francisco de Paula Sanchez Gordo, Malaga, Spain
DPB1*1778:01	HG00055417	PQ881833	Histogenetics, Ossining, USA
DPB1*1779:01	HG00055418	PQ881834	Histogenetics, Ossining, USA
DPB1*1780:01	HG00055420	PV037248	Histogenetics, Ossining, USA
DPB1*1781:01	DG10157	PQ635115	Dr Wen Jizhi, Guanghzou, China
DPB1*1782:01	s12l44j10	PQ876352	Miss Sarah Johnson, Itasta, USA
DPB1*1783:01	G 4542	PV008120	Dr Thibault Pajot, Lille, France
DPB1*1784:01	2410956070	PV067295	Dr Jonathan Visentin, Bordeaux, France
DPB1*1785:01	HG00055450	PV138035	Histogenetics, Ossining, USA
DPB1*1786:01N	HG00055451	PV138036	Histogenetics, Ossining, USA
DMA*01:01:01:44	AN303349, AN303550	OZ217107, OZ217113	Anthony Nolan Research Institute, London, United Kingdom
DMA*01:01:01:45	AN303587	OZ222274	Anthony Nolan Research Institute, London, United Kingdom
DMA*01:01:01:46	AN303589	OZ222276	Anthony Nolan Research Institute, London, United Kingdom
DMA*01:01:01:47	AN303584	OZ222271	Anthony Nolan Research Institute, London, United Kingdom
DMA*01:01:01:48	AN303586	OZ222273	Anthony Nolan Research Institute, London, United Kingdom
DMA*01:01:01:49	AN303588	OZ222275	Anthony Nolan Research Institute, London, United Kingdom
DMA*01:01:01:50	AN303585	OZ222272	Anthony Nolan Research Institute, London, United Kingdom
DMA*01:01:01:51	AN300765	OZ222262	Anthony Nolan Research Institute, London, United Kingdom
DMA*01:01:01:52	AN303582	OZ222269	Anthony Nolan Research Institute, London, United Kingdom
DMA*01:01:01:53	AN303590	OZ222277	Anthony Nolan Research Institute, London, United Kingdom
DMA*01:01:01:54	AN303583	OZ222270	Anthony Nolan Research Institute, London, United Kingdom
DMA*01:02:01:10	AN303548, AN303557, AN303563	OZ217111, OZ217120, OZ217126	Anthony Nolan Research Institute, London, United Kingdom
DMA*01:02:01:11	AN303525	OZ217109	Anthony Nolan Research Institute, London, United Kingdom
DMA*01:03:01:03	DKMS‐LSL_ID19864701, DKMS‐LSL_ID19864722	OZ003767, OZ003768	DKMS Life Sciences Lab., Dresden, Germany
DMB*01:01:01:61	AN303564, AN303568, AN303569	OZ217127, OZ217131, OZ217132	Anthony Nolan Research Institute, London, United Kingdom
DMB*01:01:01:62	AN300777	OZ222264	Anthony Nolan Research Institute, London, United Kingdom
DMB*01:01:01:63	AN303592	OZ222279	Anthony Nolan Research Institute, London, United Kingdom
DMB*01:01:01:64	AN303284	OZ222266	Anthony Nolan Research Institute, London, United Kingdom
DMB*01:01:01:65	AN01031	OZ222260	Anthony Nolan Research Institute, London, United Kingdom
DMB*01:01:01:66	AN303591	OZ222278	Anthony Nolan Research Institute, London, United Kingdom
DMB*01:02:01:06	AN300502, AN303554	OZ217103, OZ217117	Anthony Nolan Research Institute, London, United Kingdom
DMB*01:03:01:24	AN303550	OZ222268	Anthony Nolan Research Institute, London, United Kingdom
DMB*01:03:01:25	AN303552, AN303567	OZ217115, OZ217130	Anthony Nolan Research Institute, London, United Kingdom
DMB*01:03:01:26	AN303565, AN303566	OZ217128, OZ217129	Anthony Nolan Research Institute, London, United Kingdom
DMB*01:03:01:27	AN303560, AN303571	OZ217123, OZ217134	Anthony Nolan Research Institute, London, United Kingdom
DMB*01:07:01:07	AN303540, AN303555	OZ217110, OZ217118	Anthony Nolan Research Institute, London, United Kingdom
DMB*01:07:01:08	AN303593	OZ222280	Anthony Nolan Research Institute, London, United Kingdom
DOA*01:01:01:14	AN303595	OZ222282	Anthony Nolan Research Institute, London, United Kingdom
DOA*01:01:02:61	AN303597	OZ222284	Anthony Nolan Research Institute, London, United Kingdom
DOA*01:01:02:62	AN303596	OZ222283	Anthony Nolan Research Institute, London, United Kingdom
DOA*01:01:04:15	AN303599	OZ222286	Anthony Nolan Research Institute, London, United Kingdom
DOA*01:01:04:16	AN303600	OZ222287	Anthony Nolan Research Institute, London, United Kingdom
DOA*01:01:05:02	AN300768	OZ222263	Anthony Nolan Research Institute, London, United Kingdom
DOA*01:01:13	AN303594	OZ222281	Anthony Nolan Research Institute, London, United Kingdom
DOA*01:01:14	AN303598	OZ222285	Anthony Nolan Research Institute, London, United Kingdom
DOA*01:10:01:02	AN303601	OZ222288	Anthony Nolan Research Institute, London, United Kingdom
DOB*01:01:01:35	AN303561, AN303558	OZ217124, OZ217121	Anthony Nolan Research Institute, London, United Kingdom
DOB*01:01:01:36	AN303609	OZ222296	Anthony Nolan Research Institute, London, United Kingdom
DOB*01:01:01:37	AN303606	OZ222293	Anthony Nolan Research Institute, London, United Kingdom
DOB*01:01:01:38	AN303548	OZ222267	Anthony Nolan Research Institute, London, United Kingdom
DOB*01:01:01:39	AN303607	OZ222294	Anthony Nolan Research Institute, London, United Kingdom
DOB*01:01:01:40	AN303012	OZ217106	Anthony Nolan Research Institute, London, United Kingdom
DOB*01:01:01:41	AN01039, AN302997	OZ222261, OZ222265	Anthony Nolan Research Institute, London, United Kingdom
DOB*01:01:01:42	AN303604	OZ222291	Anthony Nolan Research Institute, London, United Kingdom
DOB*01:01:01:43	AN303004, AN303551	OZ217105, OZ217114	Anthony Nolan Research Institute, London, United Kingdom
DOB*01:01:01:44	AN303605	OZ222292	Anthony Nolan Research Institute, London, United Kingdom
DOB*01:01:01:45	AN303608	OZ222295	Anthony Nolan Research Institute, London, United Kingdom
DOB*01:01:03:12	AN303610	OZ222297	Anthony Nolan Research Institute, London, United Kingdom
DOB*01:01:03:13	AN303556, AN303559, AN303570	OZ217119, OZ217122, OZ217133	Anthony Nolan Research Institute, London, United Kingdom
DOB*01:01:07	AN303603	OZ222290	Anthony Nolan Research Institute, London, United Kingdom
DOB*01:03:02	AN303611	OZ222298	Anthony Nolan Research Institute, London, United Kingdom
DOB*01:05:01:02	AN303612	OZ222299	Anthony Nolan Research Institute, London, United Kingdom
DOB*01:19	AN302992, AN303553	OZ217104, OZ217116	Anthony Nolan Research Institute, London, United Kingdom
DOB*01:20	AN303602	OZ222289	Anthony Nolan Research Institute, London, United Kingdom
MICA*002:01:30	D12_GS_A	PQ296827	Dr Gaurav Sharma, Chandigarh, India

**TABLE 2:** Confirmatory sequences.

HLA allele	Cell identification	Accession number	Submitting author
A*01:217	KSR130094	PQ783998	Dr Maria Loginova, Kirov, Russia
A*02:01:197	HG00018266	MH973916	Histogenetics, Ossining, USA
A*02:07:08	TW‐102	KP404443	Prof Wei Tian, Changsha, China
A*02:446	SYNPC254	PV165884	Prof Wei Tian, Changsha, China
A*02:831N	JMDP01K0179	LC858654	Dr Masahiro Satake, Tokyo, Japan
A*02:924	004552P	PQ660451	Prof Edward KL Yang, Hualien, Taiwan
A*02:950	HG00018265	MH973915	Histogenetics, Ossining, USA
A*02:951	HG00018267, HG00018268	MH973917, MH973918	Histogenetics, Ossining, USA
A*02:1212	1797436	PQ324629	Prof Maria Gerbase De Lima, Sao Paulo, Brazil
A*03:01:01:120	112404323	PP723324	Dr Francesc Rudilla, Barcelona, Spain
A*03:01:133	511319	PV008702	Mr Alessandro Pirri, Curitiba, Brazil
A*03:503	HG00055242	PQ535516	Histogenetics, Ossining, USA
A*11:330	JMDP01K0173	LC849094	Dr Masahiro Satake, Tokyo, Japan
A*11:447Q	120274	PQ671092	Dr Francisco de Paula Sanchez Gordo, Malaga, Spain
A*23:144	HG00055402, HG00055402, HG00055402	PV037251, PV037251, PV037251	Histogenetics, Ossining, USA
A*24:85	THC1006	PV008122	Prof Wei Tian, Changsha, China
A*24:226:01	GR‐EVA‐60438	PV092589	Dr Diamanto Kouniaki, Athens, Greece
A*24:649	5712401236	PV031720	Prof Faming Zhu, Hangzhou, China
A*26:177	JMDP01K0205	LC859117	Dr Masahiro Satake, Tokyo, Japan
A*29:02:01:40	202414817	PQ811893	Dr Amalia Tejeda Velarde, Salamanca, Spain
A*29:175Q	1796634	PQ382547	Prof Maria Gerbase De Lima, Sao Paulo, Brazil
A*29:194	1816091	PQ466176	Prof Maria Gerbase De Lima, Sao Paulo, Brazil
A*30:147	HG00018744	MK032383	Histogenetics, Ossining, USA
A*31:54	120279	PQ657866	Dr Francisco de Paula Sanchez Gordo, Malaga, Spain
A*31:101	JMDP01K0441	LC859577	Dr Masahiro Satake, Tokyo, Japan
A*32:28	KSR191841	PQ783999	Dr Maria Loginova, Kirov, Russia
A*33:275	HG00055357	PQ820454	Histogenetics, Ossining, USA
A*74:01:01:02	HG00016012	MH173496	Histogenetics, Ossining, USA
B*07:02:01:39	24405046	PV037739	Dr Luis A Marin, Albacete, Spain
B*07:02:103	42441113	PQ858700	Dr Romain Ferru‐Clement, Poitiers, France
B*07:45	NT00623	PQ753128	Dr Jennifer Ng, Rockville, USA
B*07:52	NT00698	PQ787223	Dr Jennifer Ng, Rockville, USA
B*07:54	NT00718	PQ768525	Dr Jennifer Ng, Rockville, USA
B*07:137	202404019	PQ305753	Dr Amalia Tejeda Velarde, Salamanca, Spain
B*07:263	RMU4402	PQ757656	Mr Valery Cheranev, Moscow, Russia
B*08:01:11	NT01160	PQ892230	Dr Jennifer Ng, Rockville, USA
B*08:26:01	NT00570	PQ753122	Dr Jennifer Ng, Rockville, USA
B*15:01:11	NT01069	PQ892226	Dr Jennifer Ng, Rockville, USA
B*15:18:01:01	201_01390_2	PQ217970	Miss Yue Han, Xi'an, China
B*15:88	476538	PQ778460	Prof Edward KL Yang, Hualien, Taiwan
B*15:98	NT00523	PQ724295	Dr Jennifer Ng, Rockville, USA
B*15:105	NT00611	PQ753127	Dr Jennifer Ng, Rockville, USA
B*15:119	NT00675	PQ768519	Dr Jennifer Ng, Rockville, USA
B*15:164	NT01070	PQ874166	Dr Jennifer Ng, Rockville, USA
B*15:190N	RMU2098	PQ757655	Mr Valery Cheranev, Moscow, Russia
B*15:222	504887	PQ483448	Mr Alessandro Pirri, Curitiba, Brazil
B*15:596N	HG00049780, HG00049781	ON012541, ON012542	Histogenetics, Ossining, USA
B*27:04:03	NT01073	PQ874164	Dr Jennifer Ng, Rockville, USA
B*27:27	NT00526	PQ724297	Dr Jennifer Ng, Rockville, USA
B*27:37	NT00686	PQ768521	Dr Jennifer Ng, Rockville, USA
B*27:247	AB9783, AB9785	PV105079, PV105078	Prof Edward KL Yang, Hualien, Taiwan
B*27:283	HG00052628	PQ212774	Histogenetics, Ossining, USA
B*35:01:17	KSR156818	PQ784000	Dr Maria Loginova, Kirov, Russia
B*35:08:03	NT00699	PQ787224	Dr Jennifer Ng, Rockville, USA
B*35:51:01	JMDP36K0558	LC859578	Dr Masahiro Satake, Tokyo, Japan
B*35:59:01	NT00627	PQ753131	Dr Jennifer Ng, Rockville, USA
B*35:62	NT00687	PQ768522	Dr Jennifer Ng, Rockville, USA
B*35:74	NT00704	PQ787226	Dr Jennifer Ng, Rockville, USA
B*35:367:01	HG00014102	MG701522	Histogenetics, Ossining, USA
B*35:581	LIB_2467575R	PV243866	Ms Anaregina Araujo, Teresina, Brazil
B*37:01:02	NT00628	PQ753129	Dr Jennifer Ng, Rockville, USA
B*38:01:02	NT00650	PQ768517	Dr Jennifer Ng, Rockville, USA
B*38:13	NT00652	PQ768516	Dr Jennifer Ng, Rockville, USA
B*38:16	NT00710	PQ787222	Dr Jennifer Ng, Rockville, USA
B*39:01:06	2410847748	PQ860819	Dr Jonathan Visentin, Bordeaux, France
B*39:07	NT01081	PQ874165	Dr Jennifer Ng, Rockville, USA
B*39:144	HG00020128	MK165879	Histogenetics, Ossining, USA
B*40:135	NT01189	PQ892232	Dr Jennifer Ng, Rockville, USA
B*40:589	21QB002516	OZ121612	Dr Janette Kwok, Pokfulam Road, Hong Kong
B*40:592	HN2410	PQ412960	Dr Zheng Liu, Zhengzhou, China
B*42:38	0124331005959	PQ682515	Mrs Hanan Al Anazi, Riyadh, Saudi Arabia
B*44:02:04	NT00681	PQ724296	Dr Jennifer Ng, Rockville, USA
B*44:02:08	NT01072	PQ787231	Dr Jennifer Ng, Rockville, USA
B*44:03:01:35	24820307	PQ998757	Dr Luis A Marin, Albacete, Spain
B*44:44	NT00607	PQ724294	Dr Jennifer Ng, Rockville, USA
B*46:10	NT00708	PQ787227	Dr Jennifer Ng, Rockville, USA
B*47:14	HG00055360	PQ778464	Histogenetics, Ossining, USA
B*48:01:01:05	P3L2401060	PQ196804	Ms Wenqian Song, Dalian, China
B*48:15	NT00654	PQ768518	Dr Jennifer Ng, Rockville, USA
B*51:01:06	NT00525	PQ753123	Dr Jennifer Ng, Rockville, USA
B*51:60	NT01010	PQ724288	Dr Jennifer Ng, Rockville, USA
B*53:13	NT00734	PQ768520	Dr Jennifer Ng, Rockville, USA
B*54:21	476116	PV105081	Prof Edward KL Yang, Hualien, Taiwan
B*55:17	NT00530	PQ724292	Dr Jennifer Ng, Rockville, USA
B*55:140	2024TB08325	PQ243282	Dr Keming Du, Shanghai, China
B*57:01:03	NT00590	PQ753130	Dr Jennifer Ng, Rockville, USA
B*57:10	NT00604	PQ753125	Dr Jennifer Ng, Rockville, USA
B*57:24	NT01068	PQ874169	Dr Jennifer Ng, Rockville, USA
B*57:25	NT01066	PQ874171	Dr Jennifer Ng, Rockville, USA
B*58:04	NT01149	PQ892231	Dr Jennifer Ng, Rockville, USA
C*01:12:01	NT01124	PV022078	Dr Jennifer Ng, Rockville, USA
C*01:26	NT01038	PV022082	Dr Jennifer Ng, Rockville, USA
C*01:28	NT01059	PV022075	Dr Jennifer Ng, Rockville, USA
C*03:60	NT01056	PV022076	Dr Jennifer Ng, Rockville, USA
C*03:86	474284	PQ671695	Prof Edward KL Yang, Hualien, Taiwan
C*03:187	476730	PQ778462	Prof Edward KL Yang, Hualien, Taiwan
C*03:456	510939	PQ787167	Mr Alessandro Pirri, Curitiba, Brazil
C*03:693	KSR207475	PQ845330	Dr Maria Loginova, Kirov, Russia
C*03:698	KSR152011	PQ845329	Dr Maria Loginova, Kirov, Russia
C*04:01:11	NT00114	PV022072	Dr Jennifer Ng, Rockville, USA
C*04:17	NT00593	PV022073	Dr Jennifer Ng, Rockville, USA
C*04:531	HG00055337	PQ736486	Histogenetics, Ossining, USA
C*05:01:01:69	HG00030878	MZ967134	Histogenetics, Ossining, USA
C*05:37	AB9780	PV105080	Prof Edward KL Yang, Hualien, Taiwan
C*06:32	NT01121	PV022077	Dr Jennifer Ng, Rockville, USA
C*06:399	HG00055372	PQ801763	Histogenetics, Ossining, USA
C*07:73:01	NT01060	PV022080	Dr Jennifer Ng, Rockville, USA
C*07:141:01	NT01143	PV022079	Dr Jennifer Ng, Rockville, USA
C*07:997	BJLX‐1	PQ801840	Dr Xue Lian, Beijing, China
C*07:1159	SIMBHPQ768514	PQ768514	Dr Renata Monti Rocha, Belo Horizonte, Brazil
C*08:160	JMDP01K0209	LC859254	Dr Masahiro Satake, Tokyo, Japan
C*08:290	5712402195	PV031722	Prof Faming Zhu, Hangzhou, China
DRB1*03:19	6331500412	PQ811753	Dr Zhanhui Du, Qingdao, China
DRB1*04:90	6331100368	PQ811754	Dr Zhanhui Du, Qingdao, China
DRB1*04:386	HG00055461	PV174113	Histogenetics, Ossining, USA
DRB1*08:02:03	CSR0229	PQ868295	Dr Cathi Murphey, San Antonio, USA
DRB1*08:32	22N01156	PQ882774	Mrs Yanbo Nie, Tianjin, China
DRB1*13:01:19	OT02019	PQ868296	Dr Cathi Murphey, San Antonio, USA
DRB1*13:97:01	6331300120	PV208186	Dr Zhanhui Du, Qingdao, China
DRB1*14:28	JH0160	PV066008	Dr Maria Bettinotti, Baltimore, USA
DRB1*14:84	6331300307	PV208187	Dr Zhanhui Du, Qingdao, China
DRB3*02:133	NGR0077	OR885931	Dr Laura Bungener, Groningen, The Netherlands
DRB4*01:119	2455873	PV012716	Dr Bobbie Rhodes‐Clark, Mather, USA
DQA1*01:01:14	HG00055422	PQ881829	Histogenetics, Ossining, USA
DQA1*01:01:14	BMD8721	PQ868298	Dr Cathi Murphey, San Antonio, USA
DQA1*05:28	UBH0014	PQ586429	Mr Marc Kleiser, Basel, Switzerland
DQB1*05:32	2024TB10562	PQ304862	Dr Keming Du, Shanghai, China
DQB1*06:02:49	D392795	PQ778461	Prof Edward KL Yang, Hualien, Taiwan
DQB1*06:47	5312000278	PQ790246	Dr Zhanhui Du, Qingdao, China
DQB1*06:52	24405108	PV037736	Dr Luis A Marin, Albacete, Spain
DQB1*06:255	4174307	PQ872710	Dr Antonio Balas, Madrid, Spain
DQB1*06:423N	DKMS‐LSL_ID21418558	OZ210321	DKMS Life Sciences Lab., Dresden, Germany
DQB1*06:490	LIB_6019678, LIB_6180025	PQ346813, PQ346809	Ms Anaregina Araujo, Teresina, Brazil
DQB1*06:494	KSR113929	PQ665511	Dr Maria Loginova, Kirov, Russia
DQB1*06:513	HG00051084	OQ867234	Histogenetics, Ossining, USA
DQB1*06:520	021641382	PV037254	Mrs Ornella Senoussi, La Tronche, France
DQB1*06:524	BRCBC777	PV175889	Ms DongMei Li, Beijing, China
DQB1*03:01:01:05	201_00754	PQ409244	Ms Yuanli Zhao, Xi'an, China
DQB1*03:01:01:65	UERJ‐C20899	PV091011	Dr Romulo Vianna, Rio de Janeiro, Brazil
DQB1*03:01:01:71	PSCDA0799	PQ595898	Dr Seik‐Soon Khor, Tokyo, Japan
DQB1*03:02:01:29	24404932	PV015024	Dr Luis A Marin, Albacete, Spain
DQB1*03:106	111328548	PP723323	Dr Francesc Rudilla, Barcelona, Spain
DQB1*03:500:02	24‐2664	PV031333	Dr M Rosa Moya‐Quiles, El Palmar, Spain
DQB1*03:570	600785117	PQ869649	Dr Michele Curcio, Pisa, Italy
DQB1*04:02:01:22	24404883	PQ790156	Dr Luis A Marin, Albacete, Spain
DPA1*01:03:59	202421272	PQ855792	Dr Amalia Tejeda Velarde, Salamanca, Spain
DPA1*01:04:01:02	24820248	PQ874181	Dr Luis A Marin, Albacete, Spain
DPA1*01:05	1043538454	OZ206347	Dr Thomas Goeury, Geneva, Switzerland
DPA1*01:61	HN2413	PQ412962	Dr Zheng Liu, Zhengzhou, China
DPA1*01:200	TTI5527	PQ787485	Dr Cathi Murphey, San Antonio, USA
DPA1*01:203	MC012DPA1v	PV197260	Dr Zeying Du, Jacksonville, USA
DPA1*01:223Q	JH0157	PQ682504	Dr Maria Bettinotti, Baltimore, USA
DPA1*02:01:01:13	201‐00533	PP961275	Ms Yuanli Zhao, Xi'an, China
DPA1*02:01:30	1042172361	OZ206348	Dr Thomas Goeury, Geneva, Switzerland
DPA1*02:02:02:18	A6L2401442	PQ784964	Ms Wenqian Song, Dalian, China
DPA1*02:43	HG00050311	ON987391	Histogenetics, Ossining, USA
DPA1*02:151	HN2414	PQ412963	Dr Zheng Liu, Zhengzhou, China
DPB1*01:01:14	DKMS‐LSL_ID21455401	OZ211637	DKMS Life Sciences Lab., Dresden, Germany
DPB1*02:01:22	HG00025403, HG00025404	MH974010, MH974011	Histogenetics, Ossining, USA
DPB1*04:01:82	HG00055379	PQ820461	Histogenetics, Ossining, USA
DPB1*05:01:01:11	201‐00386	PQ663260	Miss Yue Han, Xi'an, China
DPB1*104:01:08	HG00051147	OR037249	Histogenetics, Ossining, USA
DPB1*625:01:02	DKMS‐LSL_ID20791218	OZ211617	DKMS Life Sciences Lab., Dresden, Germany
DPB1*687:01	JH0158	PV037685	Dr Maria Bettinotti, Baltimore, USA
DPB1*894:01N	HG00021524	MK616638	Histogenetics, Ossining, USA
DPB1*1119:01	BMD8625	PQ787484	Dr Cathi Murphey, San Antonio, USA
DPB1*1216:01	DKMS‐LSL_ID20715615	OZ211615	DKMS Life Sciences Lab., Dresden, Germany
DPB1*1325:01N	DKMS‐LSL_ID20887343	OZ211628	DKMS Life Sciences Lab., Dresden, Germany
DPB1*1348:01	DKMS‐LSL_ID21448823	OZ211621	DKMS Life Sciences Lab., Dresden, Germany
DPB1*1487:01N	DKMS‐LSL_ID20310010	OZ211613	DKMS Life Sciences Lab., Dresden, Germany
DPB1*1520:01	DKMS‐LSL_ID21639130	OZ211633	DKMS Life Sciences Lab., Dresden, Germany
DPB1*1559:01	DKMS‐LSL_ID20984835	OZ211618	DKMS Life Sciences Lab., Dresden, Germany
DPB1*1644:01	HG00055375	PQ801766	Histogenetics, Ossining, USA
DPB1*1758:01	202407020002	PQ858443	Mr Bin Chen, Tianjin, China
DMA*01:03:01:03	AN303355, AN303549, AN303572	OZ217108, OZ217112, OZ217135	Anthony Nolan Research Institute, London, United Kingdom
DOA*01:01:06	AN303562	OZ217125	Anthony Nolan Research Institute, London, United Kingdom

**TABLE 3:** Recently published sequences.

HLA allele	References
A*01:01:01:119	(Marin Rubio and Ontanon 2025c)
A*01:469	(Loginova, Dudina, and Paramonov 2025)
A*01:471	(Pajot, Elsermans, Top, and Labalette 2025a)
A*01:478	(Moya‐Quiles and Muro 2025b)
A*02:01:01:257	(Han, Liu, Wang, Feng, and Gao 2025)
A*02:01:225	(Loginova, Dudina, et al. 2025)
A*02:07:01:07	(Jing, Li, Liu, Jia, and Sun 2025)
A*02:07:08	(Tian and Li 2025)
A*02:1098	(Jiang, Xie, Ma, Hu, and Cai 2025)
A*02:1133	(X. Li et al. 2025)
A*02:1178	(Yuan, Xi, Li, Shi, and Huo 2025)
A*02:1193	(Loginova, Dudina, et al. 2025)
A*02:1195N	(Schmitt and Gothot 2025b)
A*02:1196	(Mendes‐Oliveira et al. 2025)
A*02:1198	(Pajot et al. 2025b)
A*03:01:130	(Cargou, Elsermans, Top, Wojchiechowski, and Visentin 2025a)
A*03:497	(Loginova, Dudina, et al. 2025)
A*11:01:79	(K. L. Yang and Lin 2025b)
A*11:481	(Shi et al. 2025)
A*11:485	(D. M. Li et al. 2025)
A*23:01:01:34	(Marin Rubio and Ontanon 2025c)
A*23:01:01:35	(Marin Rubio and Ontanon 2025c)
A*24:620	(Gatouillat, Salez, Libyh, Tabary, and Giusti 2025)
A*25:92	(Loginova, Dudina, et al. 2025)
A*26:252N	(Loginova, Dudina, et al. 2025)
A*29:02:01:40	(Marin Rubio and Ontanon 2025c)
A*29:200N	(Pajot, Elsermans, Top, and Labalette 2025c)
A*31:01:58	(Fan, Wen, Li, Zhang, and Sun 2025)
A*32:01:59	(Cargou et al. 2025b)
A*32:01:62	(Pajot et al. 2025d)
A*33:01:01:17	(Marin Rubio and Ontanon 2025c)
A*33:01:01:19	(Marin Rubio and Ontanon 2025c)
A*33:274	(Loginova, Dudina, et al. 2025)
A*66:49	(Loginova, Dudina, et al. 2025)
A*68:325	(Pajot, Elsermans, Top, and Labalette 2025e)
B*07:02:107	(Loginova, Obukhov, and Paramonov 2025)
B*08:01:78	(Loginova, Obukhov, et al. 2025)
B*08:331	(Rakhmanov, Bernheiden, Verboom, Hallensleben, and Emmerich 2025d)
B*13:201	(Loginova et al. 2025)
B*14:01:01:12	(Rubio et al. 2025b)
B*14:136	(Guan et al. 2025)
B*15:01:01:67	(Rubio et al. 2025b)
B*15:01:89	(Loginova, Obukhov, et al. 2025)
B*15:11:08	(J. M. Li, Lou, Ding, Luo, and Cai 2025)
B*15:245:02Q	(Xia and Li 2025)
B*15:662	(D. Chen et al. 2025)
B*18:01:56	(Cheranev et al. 2025)
B*18:248N	(Loginova et al. 2025)
B*27:05:02:44	(S. Agarwal, Kumari, Jaiswal, Kumar, and Kumar 2025)
B*27:282	(Loginova and Paramonov 2025)
B*27:284	(Schmitt and Gothot 2025a)
B*35:01:01:90	(Rubio et al. 2025b)
B*35:01:80	(Cheranev et al. 2025c)
B*35:03:01:38	(Rubio et al. 2025b)
B*35:31:01:02	(Rubio et al. 2025b)
B*35:186	(X. Zhang et al. 2025)
B*35:628	(Pajot et al. 2025f)
B*37:114	(You et al. 2025)
B*38:01:26	(Cheranev et al. 2025a)
B*38:114	(Morel, Peres, Clement, Aarnink, and Dhuyser 2025)
B*38:121	(Pajot, Elsermans, Top, and Labalette 2025g)
B*38:122	(Gonen and Demir 2025)
B*39:226	(Loginova et al. 2025)
B*40:01:82	(Gao et al. 2025)
B*40:02:39	(Dhuyser et al. 2025e)
B*40:593	(Loginova and Paramonov 2025)
B*41:01:01:09	(Rubio et al. 2025b)
B*44:02:01:85	(Rubio et al. 2025b)
B*44:384	(Dhuyser et al. 2025a)
B*44:417	(Pajot et al. 2025h)
B*51:01:01:125	(Rakhmanov et al. 2025a)
B*51:385	(Hao et al. 2025)
B*52:01:58	(Loginova, Obukhov, et al. 2025)
B*52:130	(Cheranev, Jankevich, Smirnova, Loginova, and Korostin 2025)
B*54:49N	(Loginova et al. 2025)
B*56:102	(Fournel et al. 2025)
B*58:01:50	(K. L. Yang and Lin 2025a)
B*58:01:51	(Fabreti‐Oliveira et al. 2025)
B*58:159	(Guan et al. 2025)
C*01:250	(H. Y. Yang et al. 2025)
C*01:280	(Loginova et al. 2025)
C*01:282	(H. Zhao et al. 2025)
C*01:283	(de Lima, Molinari, Massi, Hirata, and Visentainer 2025)
C*02:231	(Ingrassia, Pecoraro, Aiello, Fedele, and Cappuzzo 2025a)
C*03:09:02:01	(Fabreti‐Oliveira et al. 2025a)
C*03:657	(Kim, Lee, Kim, and Won 2025)
C*03:678	(Centuriao, Maciel, Goldenstein, Alonso, and Torres 2025)
C*03:681	(Luo et al. 2025)
C*03:689	(Y. J. Wen, Fan, Liu, Wang, and Sun 2025)
C*03:698	(Loginova et al. 2025)
C*03:699	(Loginova et al. 2025)
C*03:700	(Loginova et al. 2025)
C*03:702	(Cheranev et al. 2025a)
C*04:533	(Ingrassia et al. 2025b)
C*04:534	(Blandin et al. 2025)
C*05:01:83	(Pajot, Elsermans, Top, and Labalette 2025i)
C*05:303N	(Loginova et al. 2025)
C*06:44:02	(Jovanovic et al. 2025)
C*07:04:32	(Fabreti‐Oliveira et al. 2025a)
C*07:1102	(Dhuyser, Peres, Morel, Clement, and Aarnink 2025f)
C*07:1130N	(Dhuyser, Peres, Morel, Clement, and Aarnink 2025b)
C*07:1145N	(Pinto, Santos, Vianna, Porto, and Secco 2025)
C*07:1152	(Fabreti‐Oliveira et al. 2025a)
C*07:1172	(Rakhmanov et al. 2025c)
C*08:300	(Fabreti‐Oliveira et al. 2025a)
C*08:301	(M. Chen, Yisimayili, Xu, Zhang, and Tong 2025)
C*12:02:55	(Cheranev, Evgrafova, Smirnova, Loginova, and Korostin 2025b)
C*12:428N	(Loginova et al. 2025)
C*14:02:41	(Lee and Choi 2025)
C*15:02:01:65Q	(Pinto et al. 2025)
C*15:02:01:69Q	(Rakhmanov, Bernheiden, Verboom, Hallensleben, and Emmerich 2025b)
C*15:275	(Dhuyser et al. 2025c)
DRB1*03:72:02	(D. Zhang et al. 2025)
DRB1*07:159	(Dhuyser, Peres, Morel, Clement, and Aarnink 2025d)
DRB1*08:130	(Marin Rubio and Ontanon 2025a)
DRB1*11:337	(Jimenez et al. 2025)
DRB1*11:340	(Blandin, Visentin, Dalhoumi, Rouzaire, and Lemal 2025)
DRB1*13:01:44	(Pajot, Elsermans, Top, and Labalette 2025m)
DRB1*13:348	(Dhuyser, Peres, Morel, Clement, and Aarnink 2025l)
DRB3*02:213	(Bouthemy et al. 2025)
DRB4*01:03:01:22	(Y. Zhao et al. 2025)
DRB5*01:02:05	(Pajot, Lion, Elsermans, Guillaume, and Desoutter 2025)
DQA1*01:67:02	(Cargou, Andreani, Gustiniani, Ralazamahaleo, and Visentin 2025)
DQA1*01:144	(Velickovic et al. 2024)
DQA1*01:145	(Velickovic et al. 2024)
DQA1*01:146	(Velickovic et al. 2024)
DQA1*01:147	(Velickovic et al. 2024)
DQA1*01:157	(Pajot et al. 2025)
DQA1*02:01:23	(Velickovic et al. 2024)
DQA1*02:39	(Dhuyser, Peres, Morel, Clement, and Aarnink 2025h)
DQA1*03:69	(Velickovic et al. 2024)
DQA1*03:70	(Velickovic et al. 2024)
DQA1*04:19	(Velickovic et al. 2024)
DQA1*05:05:24	(Lion et al. 2025)
DQA1*05:89	(Dhuyser et al. 2025i)
DQA1*05:90	(Gaballa, Soderstrom, Lundin, and Eich 2025)
DQA1*05:102	(Velickovic et al. 2024)
DQA1*05:118:01:01	(Ocejo‐Vinyals, Mota‐Perez, Garcia, Toriello, and Balas 2025)
DQB1*05:03:38	(Quan and Cai 2025)
DQB1*05:311:02	(Pajot et al. 2025j)
DQB1*05:327	(Velickovic et al. 2024)
DQB1*05:328	(Velickovic et al. 2024)
DQB1*05:351	(Bamrdouf et al. 2025)
DQB1*05:354	(Pajot, Elsermans, Top, and Labalette 2025k)
DQB1*05:355	(Pajot et al. 2025l)
DQB1*06:02:60	(Dhuyser et al. 2025k)
DQB1*06:02:65	(Cheranev, Jankevich, Makhova, Loginova, and Goltsov 2025b)
DQB1*06:03:01:23Q	(Velickovic et al. 2024)
DQB1*06:304N	(Liacini and Geier 2025)
DQB1*06:469	(Muniyappa et al. 2025)
DQB1*06:482	(Velickovic et al. 2024)
DQB1*06:524	(D. Zhang, Wang Liu, Sun, and Wang 2025)
DQB1*02:01:01:27	(Y. Li, Zhao, Han, Chen, and Gao 2025)
DQB1*02:180:02	(Velickovic et al. 2024)
DQB1*02:216	(Velickovic et al. 2024)
DQB1*03:01:01:73	(Ocejo‐Vinyals et al. 2025)
DQB1*03:01:59	(Dhuyser, Peres, Morel, Clement, and Aarnink 2025j)
DQB1*03:518	(Velickovic et al. 2024)
DQB1*03:571	(Moya‐Quiles and Muro 2025a)
DQB1*03:572	(Wanyan, Bian, Wang, Zheng, and Yang 2025)
DQB1*04:104	(Santos, Mendes‐Oliveira, Vianna, Porto, and Secco 2025)
DPA1*01:03:55	(Velickovic et al. 2024)
DPA1*01:192	(Velickovic et al. 2024)
DPA1*01:193	(Velickovic et al. 2024)
DPA1*01:194	(Velickovic et al. 2024)
DPA1*01:199	(Velickovic et al. 2024)
DPA1*02:01:28	(Velickovic et al. 2024)
DPA1*02:01:34	(H. Zhao, Webster, Hammond, Abdelfatah, and Mostafa 2025)
DPA1*02:02:02:17Q	(Velickovic et al. 2024)
DPA1*02:02:02:19	(Y. Zhao, Chen, Wang, Zhu, and Ren 2025)
DPA1*02:127	(Velickovic et al. 2024)
DPB1*04:02:27	(Cargou et al. 2025)
DPB1*05:01:23	(Zhu, Xie, Zheng, Zhou, and Luo 2025)
DPB1*1483:01	(Oh et al. 2025)
DPB1*1498:01	(Lou, Xie, Ma, Hu, and Lei 2025)
DPB1*1582:01	(Velickovic et al. 2024)
DPB1*1583:01	(Velickovic et al. 2024)
DPB1*1617:01	(Dhuyser et al. 2025g)
DPB1*1619:01	(Fabreti‐Oliveira, de Oliveira, da Silva Assis, and Nascimento 2025b)
DPB1*1621:01	(Fabreti‐Oliveira, de Oliveira, et al. 2025b)
DPB1*1626:01Q	(Jimenez et al. 2025)
DPB1*1737:01	(Fabreti‐Oliveira, de Oliveira, et al. 2025b)
DPB1*1781:01	(J. Wen et al. 2025)
MICB*004:01:31	(D. Agarwal et al. 2025)
MICB*004:01:32	(D. Agarwal et al. 2025)
MICB*004:01:33	(D. Agarwal et al. 2025)
MICB*005:02:59	(D. Agarwal et al. 2025)

An N suffix was added to the DRB4*01:03:01:26 allele in February 2025, now making it DRB4*01:03:01:26N, as this allele was shown to have the same splice mutation as was previously reported in the DRB4*01:03:01:02N allele.

All new and confirmatory sequences should now be submitted directly to the WHO Nomenclature Committee for Factors of the HLA System via the IPD‐IMGT/HLA Database using the sequence submission tool provided (Barker et al. 2023; Robinson et al. 2024). The IPD‐IMGT/HLA Database may be accessed via the World Wide Web at: www.ebi.ac.uk/ipd/imgt/hla

## Data Availability

Data sharing not applicable to this article as no datasets were generated or analyzed during the current study.
